# Cardio-Ankle Vascular Index and Aging: Differences between CAVI and CAVI0

**DOI:** 10.3390/jcm12216726

**Published:** 2023-10-24

**Authors:** Anna Giani, Rocco Micciolo, Elena Zoico, Gloria Mazzali, Mauro Zamboni, Francesco Fantin

**Affiliations:** 1 Section of Geriatric Medicine, Department of Surgery, Dentistry, Pediatric and Gynecology, University of Verona, 37100 Verona, Italy; anna.giani@univr.it (A.G.); mauro.zamboni@univr.it (M.Z.); 2 Centre for Medical Sciences and Department of Psychology and Cognitive Sciences, University of Trento, 38123 Trento, Italy; rocco.micciolo@unitn.it; 3 Section of Geriatric Medicine, Department of Medicine, University of Verona, 37100 Verona, Italy; elena.zoico@univr.it (E.Z.); gloria.mazzali@univr.it (G.M.)

**Keywords:** arterial stiffness, arterial aging, CAVI, CAVI0, older adults

## Abstract

Background: Cardio-ankle vascular index (CAVI) and CAVI0 (a mathematical expression derived from CAVI, supposed to be less dependent on blood pressure), can describe arterial stiffness, considering a wide proportion of the arterial tree. The aim of this study was to examine the relationship between CAVI, CAVI0 and aging, looking at the differences between the two arterial stiffness indexes. Methods: A total of 191 patients (68 male, mean age 68.3 ± 14.4 years) referred to the Geriatric Ward and Outpatient Clinic at Verona University Hospital were included and underwent a comprehensive clinical evaluation. CAVI and CAVI0 were obtained for each. Results: CAVI0 steeply rises in the elderly age strata, widening the gap between CAVI and CAVI0. An inverse relationship is evident between CAVI0 and DBP in older patients, and CAVI0 is shown to be dependent on age, DBP and age-DBP interaction (R^2^ = 0.508). Age modifies the effect of DBP on CAVI0, but not on CAVI. Conclusions: The real new findings of our study are that the association between CAVI0 and diastolic blood pressure (DBP) is modified by age, whereas the association between CAVI and DBP is not modified by age. From a clinical point of view, these are very important findings, as DBP decreases with aging, affecting in elderly populations the reliability of CAVI0, which strictly depends on DBP in the formula to calculate it. To monitor the effect of CV therapies, progression of CV diseases and to evaluate clinical outcomes in elderly populations, we suggest using CAVI and not CAVI0.

## 1. Introduction

Arterial wall stiffness plays a key role in the pathophysiological mechanism of vascular aging [1] and its evaluation is of paramount importance to characterize the cardiovascular risk [2]. Several parameters have recently been described, yet less is known about which of them are more appropriate in the geriatric settings. In addition to the well-known tonometric pulse wave velocity (PWV), which is also considered the gold standard under the latest European guidelines [3], the cardio-ankle vascular index (CAVI) can provide an interesting description of arterial stiffness, aimed to include the whole arterial tree and to reduce the dependence on blood pressure [4]. First described by Shirai and colleagues [4], CAVI adjusts the PWV (calculated from aortic valve orifice to the ankle) considering both arterial wall compliance and elastic properties, and blood viscosity, providing a global evaluation of stiffness, from the aorta to the tibial arteries. Later in 2016, Spronck and colleagues suggested a new formula [5] to define CAVI0, in order to reduce the dependence of CAVI on arterial blood pressure (BP) at the time of measuring, introducing a reference pressure of 100 mmHg. Consolidated knowledge demonstrated the association between CAVI and CAVI0 [6,7]; nevertheless, the choice of CAVI instead of CAVI0 has been widely debated [5,8,9], and the real independence of CAVI0 from BP is yet to be clearly demonstrated [6,7,10,11]. Thus, there is a fair uncertainty regarding CAVI0, which might not be accurate in any subsets, and its suitability in older adults should be further examined.

What is acknowledged is that older adults display increased arterial stiffness, and accordingly, CAVI and CAVI0 are shown to be increased [6,8]. As a matter of fact, CAVI increases with age; the increasing trend is quite controversial: a 0.5 increase in CAVI every 10 years has been described [12], but other studies outlined a differential increase in different age strata, due to a nonlinear relation [13]. So far, less is known about CAVI0.

It should be noted that several cardiovascular disorders and risk factors, broadly common in older age, are known to be associated with higher CAVI: namely arterial hypertension [14,15], diabetes [12], dyslipidemia [16], coronary artery disease [17] and carotid artery plaques [18].

The aim of the present study was to compare CAVI and CAVI0 in a wide population of adults, to examine the relationship between CAVI, CAVI0 and aging, looking at the differences between the two arterial stiffness indexes.

## 2. Materials and Methods

A total of 191 subjects (119 female and 64 male), mean age 67.5 ± 14.3 years, hospitalized at the Geriatric Clinic of Verona University Hospital or referred to the outpatient clinic (medical nutrition or arterial hypertension) formed the study population. Exclusion criteria were: (I) limb amputation or history of surgical treatment of the aorta or carotid or femoral arteries; (II) severe peripheral arterial disease or proximal arterial stenosis; (III) atrial fibrillation or other major arrhythmias. A comprehensive clinical evaluation was performed, including clinical history collection.

The study was approved by the Ethical Committee of the University of Verona. All participants gave informed consent to be involved in the research study.

### 2.1. Anthropometric Variables

Body weight (Salus scale, Milan, Italy) and height were recorded (Salus stadiometer, Milan, Italy), with the subject barefoot and wearing light indoor clothing. Whenever patients could not assume the erect position, the last anamnestic height was recoded. BMI was calculated as body weight adjusted by stature (kg/m^2^).

### 2.2. Biochemical Analyses

All patients received venous blood sampling, after overnight fasting. Plasma glucose was measured with a glucose analyzer (Roche Cobas 8000, Monza, Italy). Cholesterol and triacylglycerol concentrations were determined with spectrophotometric method (Roche Cobas 8000, Monza, Italy). High-density-lipoprotein (HDL) cholesterol was measured by using the method of Warnick and Albers. LDL cholesterol was calculated using the Friedwald formula. Creatinine was measured by a modular analyzer (Roche Cobas 8000, Monza, Italy).

### 2.3. Blood Pressure and Arterial Stiffness Measurements

As we previously described [19], VaSera-1500 (Fukuda-Denshi Company, Ltd., Tokyo, Japan) was used to obtain CAVI, blood pressure and heart rate; the same device provides mean arterial pressure (MAP) and pulse pressure (PP). BP cuffs were placed simultaneously on the four limbs and inflated two by two (right and left side) to increase the accuracy of measurements. ECG was obtained by two electrodes placed on both arms; to obtain phonocardiography, a microphone was placed on the sternum (second rib space). CAVI derives from the Bramwell-Hill Formula [20,21], which is based on heart-ankle PWV, obtained by the following equation:

(1) $$CAVI=a*\left(ln\frac{SBP}{DBP}*\frac{{PWV}^{2}*2\rho }{SBP-DBP}\right)+b$$

where a and b are constants, and ρ is considered the blood density. The device can directly provide heart-ankle PWV (haPWV) as the ratio between aortic valve to ankle length and the time T, where T stands for tb + tba, taken by pulse wave to run this distance (tb: time from the second heart sound to the dicrotic notch at the brachial pulse wave form, tba: time from brachial to ankle pulse waves) [22]. A brachial-ankle PWV (baPWV) can be eventually derived [23]. CAVI0 was derived by proper electronic calculator [24] following the formula:

(2) $$CAVI0=\frac{CAVI-b}{a}*\frac{\frac{SBP}{DBP}-1}{\ln\left(\frac{SBP}{DBP}\right)}-ln\left(\frac{SBP}{P_{ref}}\right)$$

and considering P_ref_ as a standard pressure of 100 mmHg.

### 2.4. Statistical Analyses

The results are shown as mean value ± standard deviation (SD). Pearson correlation coefficient was used to estimate associations between variables. Linear multiple regression analysis was employed to evaluate the effect of age and DBP on CAVI and CAVI0, taking into account the effect of other selected variables (SBP, sex and BMI). Analysis of variance (ANOVA) was performed to evaluate the effect of independent variables included in regression models. Among the considered variables, CAVI0, CAVI, height and SBP showed a normal quantile plot giving some evidence for a difference from the normal distribution. However, the same result was found when logarithmic, square root and reciprocal transformations were employed. Furthermore, computer simulations showed that sample means based on samples of about 100 observations (like those presented in this study) can be considered normally distributed. However, when comparing mean values from two samples of subjects, in addition to the standard Student’s *t* test for unpaired data, the non-parametric Mann-Whitney U test was employed.

A significance threshold level of 0.05 was used throughout the study. All statistical analyses were performed using R (version 4.2.2, R Core Team (2022)), a language and environment for statistical computing (R Foundation for Statistical Computing, Vienna, Austria; https://www.R-project.org/).

Residuals of regression analyses were visually checked for normality employing a normal quantile plot. Although the tails of the distribution points did not lie close to a straight line, the pattern was symmetric. The significance of the results was also checked employing a distribution free permutation test for regression models implemented in the R package “lmPerm” (Wheeler, B.; Torchiano, M. lmPerm: Permutation Tests for Linear Models; R package version 2.1.0, 2016; https://CRAN.R-project.org/package=lmPerm (accessed on 18 October 2023)).

## 3. Results

Data were considered for a total of 191 patients (68, 35.6%, male). Their ages ranged between 40 and 96 years (mean age 68.3 ± 14.4 years; median age 69 years). DBP ranged between 55 and 109 mmHg (mean DBP 81.2 ± 10.7 mmHg; median DBP 81 mmHg). The main characteristics of the population, subdivided using an age threshold of 70 years (100 subjects < 70 years, 91 subjects ≥ 70 years), are listed in Table 1. All the mean values (except height, SBP, glucose levels and tryglicerides) of these two samples showed highly significant differences both when the Student’s *t*-test for unpaired data and the non-parametric Mann-Whitney U test were employed.

As compared to the younger subgroup, older patients (≥70 years) had significantly lower DBP (mean 76.51 ± 10.21 mmHg vs. 85.4 ± 9.35 mmHg *p* < 0.001) and MAP (97.2 ± 13.71 mmHg vs. 105.4 ± 10.64 mmHg, *p* < 0.001). Higher CAVI (10.25 ± 2.15 vs. 7.78 ± 1.21, *p* < 0.001) and CAVI0 (18.9 ± 6.64 vs. 11.49 ± 2.64, *p* < 0.001) were described in the oldest subgroup. As concerns the anthropometric variables, older subjects had both lower BMI (25.84 ± 5.68 kg/m^2^ vs. 31.38 ± 4.82 kg/m^2^ *p* < 0.001) and lower waist circumference (97.18 ± 15.19 cm vs. 103.6 ± 13.88 cm, *p* = 0.006) than younger patients.

No significant difference was detected in glucose and triglycerides levels between groups; on the other hand, older patients had reduced total cholesterol (150.4 ± 3831 mg/dL vs. 202.8 ± 41.42 mg/dL, *p* < 0.001), LDL cholesterol (70.8 ± 35.16 mg/dL vs. 123.2 ± 35.54 mg/dL, *p* < 0.001) and HDL cholesterol (45.5 ± 17.15 mg/dL vs. 55.5 ± 15.42 mg/dL, *p* < 0.001).

Cardiovascular risk factors have also been considered: arterial hypertension was significantly more prevalent in older subjects (*p* = 0.02), whereas dyslipidemia was significantly more prevalent in younger patients (*p* = 0.031). Any significant difference was detected when looking at smoking habits and diabetes prevalence.

**Figure 1:**
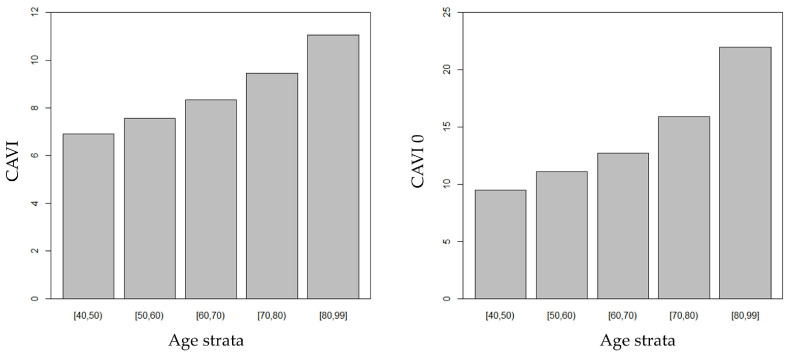
CAVI and CAVI0 increased through consecutive age strata.

A significant negative association was found between DBP and age (r = −0.464, *p* < 0.001). CAVI and CAVI0 progressively increased through consecutive age strata (Figure 1), with a significant trend even after adjustment for DBP. Noteworthily, CAVI0 steeply increased after the age threshold of 70 years, therefore increasing the gap between CAVI and CAVI0.

**Figure 2:**
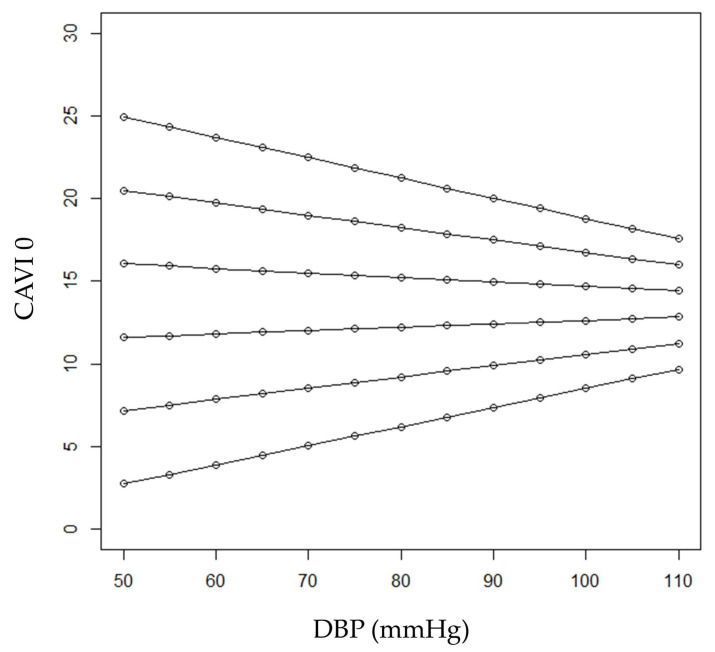
Predicted CAVI0 values in relation to DBP for selected ages (from bottom to top: 40, 50, 60, 70, 80 and 90 years).

CAVI0 was significantly associated both with age (r = 0.703; *p* < 0.001) and, negatively, with DBP (−0.360; *p* < 0.001). Noteworthily, when CAVI0 was considered as the dependent variable in a regression model, a significant interaction between age and DBP was found (*p* = 0.027), revealing that the relationship between CAVI0 and DBP was modified by age. Figure 2 shows predicted CAVI0 values in relation to DBP for selected ages. Predicted values were calculated employing the estimates of the regression coefficients shown in Table 2.

In younger ages (40 and 50 years, the first three lines from bottom in the figure), subjects with higher DBP were expected to have higher CAVI0 values. On the other hand, in older patients, an inverse relationship between CAVI0 and DBP was expected (see the first two lines from the top in the figure, referring to patients aged 90 and 80). Expected CAVI0 values for 60-year-old patients range from 11.6 to 12.8 when DBP values varied between 50 and 110 mm Hg, respectively. On the other hand, expected CAVI0 values for 70-year-old patients ranged from 16.0 to 14.4 when DBP values varied between 50 and 110 mmHg, respectively. At the age of (about) 64 years, CAVI0 was expected to be constant (i.e., independent from DBP) at the value of 13.5.

The regression model, which included age, DBP and the interaction between age and DBP, showed a multiple R^2^ of 0.508 (R = 0.713). Table 2 shows the estimated regression coefficients for this model, together with the corresponding standard errors.

Residuals of these two regression analyses were visually checked for normality employing a normal quantile plot, which showed a symmetric pattern of the points even if in the tails of the distribution they did not lie close to a straight line. The significance of the results was also checked employing a distribution-free permutation test for regression models implemented in the R package “lmPerm”.

When SBP, sex and BMI were included as independent variables in the regression model, the interaction between age and DBP maintained the statistical significance (*p* = 0.025). In addition, when this model was considered, the effects of SBP and BMI were also statistically significant (with a negative association for BMI); the R^2^ of this model was 0.530 (R = 0.728). Table 3 shows the estimated regression coefficients for this model, together with the corresponding standard errors.

CAVI was significantly associated both with age (r = 0.683; *p* < 0.001) and, negatively, with DBP (−0.266; *p* < 0.001). However, when CAVI was considered the dependent variable in a multiple regression model, only age was significantly associated with the response (*p* = 0.01), while DBP as well as the interaction between age and DBP were not significant (*p* = 0.181 and *p* = 0.259, respectively). Table 4 shows the estimated regression coefficients for this model, together with the corresponding standard errors.

A similar result was found when the interaction term was removed from the previous model. Therefore, when the effect of age was accounted for, the correlation between CAVI and DBP was no longer significant and only age remained significantly associated with CAVI.

When SBP, sex and BMI were included as independent variables in the regression model, the interaction between age and DBP confirmed it was not a significant result (*p* = 0.246). Furthermore, when this model was considered, DBP (*p* = 0.265) and SBP (*p* = 0.633) were not significantly associated with CAVI, while a significant effect of both BMI (*p* = 0.005) and sex (*p* = 0.019) was found. The R^2^ of this model was 0.511 (R = 0.715). Table 5 shows the estimated regression coefficients for this model, together with the corresponding standard errors.

Residuals of these two regression analyses were visually checked for normality employing a normal quantile plot, which showed a symmetric pattern of the points even if in the tails of the distribution they did not lie close to a straight line. The significance of the results was also checked employing a distribution-free permutation test for regression models implemented in the R package “lmPerm” (version 2.1.0, 2016).

Therefore, when considering CAVI0 and CAVI as dependent variables, two different results were found. The main difference was that age modified the effect of DBP on CAVI0, but not on CAVI. Furthermore, BMI appeared to have a significant (and negative) effect on both CAVI0 and CAVI, while sex was significant only for CAVI (with higher values for males).

## 4. Discussion

The present study, of 191 adults ranging from 40 to 96 years, shed light on the significant association between aging and arterial stiffness, measured by CAVI and CAVI0; however, our data suggest that age modifies the effect of DBP on CAVI0, but not on CAVI, opening significant perspectives on the choice of CAVI rather than CAVI0 when examining arterial stiffening in older adults.

Our study moves from the assumption that CAVI is a valid estimate of arterial stiffening in older ages [25]. Although guidelines endorse the use of cfPWV as the gold standard for arterial stiffness evaluation [3], there has been increasing interest in CAVI and CAVI0 [26]. As compared to cfPWV, which is a particular measurement of the central aortic segments, CAVI is known to be representative of a wider proportion of the arterial tree, including both central and peripheral segments [13]. Owing to this intrinsic property of the technique, in older adults, CAVI rather than cfPWV might be considered more effective in highlighting the hallmarks of aging-related pathophysiological changings; we previously demonstrated [19] a significant relationship between arterial stiffness indexes and age, showing that the strength of the association is higher for CAVI and CAVI0, as compared to cfPVW.

CAVI0 derives from CAVI with the main aim of relieving the residual pressure dependency that was still found in CAVI [27]. In Shirai’s equation [4], in fact, CAVI relies on a stiffness parameter β, which depends on the arterial pressure and on the vessel diameter, following the equation:

(3) $$\beta =\ln\frac{SBP}{DBP}*\frac{D}{∆D}$$

where D stands for the vessel diameter and ∆D stands for its changing; thus, β is not a pressure-normalized index. On the other hand, due to the introduction of a unique P_ref_ (proposed to be equal to 100 mmHg) [24,27], CAVI0 is based on a β0 parameter, and it is considered to be pressure normalized:

(4) $$\beta 0=\beta -\ln\frac{DBP}{P_{ref}}$$

Several features are common to CAVI and CAVI0, namely the included arterial segments (the entire arterial tree from the origin of the aorta to the ankle), the BP measurement site at the upper brachial artery and the baPWV, which accounts for the total measured artery [27,28]. Nonetheless, a major difference is pinpointed when looking at the formulas: it should be noted that CAVI depends on a mid-pressure (the arithmetic mean between DBP and SBP, see Equation (1)), whereas CAVI0 depends on DBP [29] following an inverse relation [5]:

(5) $$CAVI0=2\rho *\frac{{PWV}^{2}}{DBP}-\frac{DBP}{P_{ref}}$$

This consideration is a trivial point to interpret the relationship connecting age, CAVI and CAVI0.

We observed that in younger ages, subjects with higher DBP were expected to have higher CAVI0 values, which is in line with previous findings by Webb and colleagues [30], who demonstrated that midlife DBP is a significant predictor of arterial stiffness and progression of arterial stiffness. Authors provided evidence that higher DBP during midlife is associated with an earlier transition from a rising to a falling DBP [30], reflecting a well-known mechanism of arterial aging which results in greater arterial stiffness and lower DBP at older ages [31]. As a matter of fact, impaired arterial compliance, due to arterial aging, is also responsible for reduced DBP among older adults. Consolidated knowledge describes isolated systolic hypertension as the most frequent phenotype in subjects aged over 50 years [32], also identifying in lower DBP a relevant risk factor for all-cause mortality [33]. Consequently, older adults are more likely to have low DBP and, mathematically, greater CAVI0.

In line with these considerations, we outlined an inverse relationship between CAVI0 and DBP in older subjects. In particular, our data showed that CAVI0 steeply increases after the age threshold of 70 years, while this was not true for CAVI.

We compared the predictors of CAVI and CAVI0, observing CAVI0 results to be strongly dependent on age, DBP and on the interaction between age and DBP. In other words, age modifies the effect of DBP on CAVI0, but not on CAVI.

Including older adults in our analyses, our results complement previous evidence, since CAVI0 has been applied in younger age sets, such as the pediatric [34] and adolescent [35] ages. So far, most of the studies led on CAVI0 compared heathy individuals versus subjects with cardiovascular disorders [6], normal weight versus overweight patients [35] and different subsets of hypertensive patients [36,37]; however, to the best of our knowledge, the possible changing of CAVI0 during aging has never been investigated.

There is a rather limited number of studies comparing CAVI and CAVI0, even taking into account the quite recent introduction of CAVI0 in research practice. Previous evidence suggested the superiority of CAVI over CAVI0 in the predictive role on atherosclerotic plaque formation [38]. Furthermore, CAVI, but not CAVI0, has been shown to be accurate in reflecting not only organic structural stiffness but also functional stiffness [12,38] and hemodynamic changes [39]. CAVI0 is deemed to underestimate arterial stiffness in subjects with high DBP [6]. In a longitudinal study of Japanese subjects, Nagayama and colleagues recently demonstrated the superior predictability of CAVI compared to PWV and CAVI0 for renal function decline [40]. However, the comparison between CAVI and CAVI0 in older adults has never been explored.

Thus, besides the agreement of both CAVI and CAVI0 in describing increased arterial stiffness among aged subjects, the suitability of CAVI0 in older age strata might be prevented by a lower DBP, which unavoidably leads to higher CAVI0.

In line with previous findings, our data suggest that BMI might have a significant and negative effect on both CAVI0 and CAVI, while sex is significant only for CAVI (with higher values for males). Previous studies demonstrated that CAVI was negatively correlated with BMI, and also with waist circumference; the result might appear counterintuitive, since higher arterial stiffness is expected in patients with obesity [41,42]. However, a recent study led by Nagayama and colleagues showed that a body shape index, instead of BMI, as proxy for visceral adiposity, was associated with CAVI increase [43].

The strengths and limitations of the present study should be recognized. This is the first study focusing on CAVI0 measurement in older adults, and its changings across aging, analyzing a heterogeneous range of age strata. On the other hand, our population was not made up of healthy subjects, and the concomitant inclusion of both inpatients and outpatients, with different clinical conditions (although all the patients were examined after achieving clinical stability), might have brought heterogeneity into the stiffness measurements. Moreover, our study was predominantly performed on female patients, and given the increased prevalence of cardiovascular diseases in the male population, associated with higher CAVI values, testing our hypothesis in a more representative male population would be beneficial.

## 5. Conclusions

In conclusion, our study, on a relatively wide and heterogeneous cohort of patients, outlines a strong association between arterial stiffness indexes and age, showing that the association between CAVI0 and diastolic blood pressure is modified by age, whereas the association between CAVI and DBP is not modified by age.

From a clinical point of view, this is a very important finding, as DBP decreases with aging, affecting in elderly populations the reliability of CAVI0, which strictly depends on DBP in the formula to calculate it.

In other words, for these reasons, in clinical practice we suggest that to monitor the effect of CV therapies, progression of CV diseases and to evaluate clinical outcomes, CAVI and not CAVI0 should be used in elderly populations.

## Figures and Tables

**Table 1:** Characteristics of the study population, divided by age strata.

	<70 Years (n = 100)					≥70 Years (n = 91)						
	Mean	SD	Median	1st q.le	3rd q.le	Mean	SD	Median	1st q.le	3rd q.le	*p*	p*
Age (years)	56.86	8.53	58.00	51.00	64.00	80.79	7.49	79.00	74.00	87.00	<0.001	<0.001
DBP (mmHg)	85.40	9.35	86.00	79.00	92.00	76.51	10.21	76.00	70.00	84.00	<0.001	<0.001
SBP (mmHg)	141.81	16.36	139.50	130.00	153.00	137.07	20.17	136.00	126.00	148.00	0.075	0.086
MAP (mmHg)	105.4	10.64	106.00	98.75	114.33	97.2	13.71	96.7	89.33	97.21	<0.001	<0.001
PP (mmHg)	54.9	13.18	53.00	46.75	60.00	60.07	14.53	59.00	50.50	67.00	0.011	0.004
CAVI0	11.49	2.64	10.91	9.58	13.44	18.90	6.64	17.00	14.09	21.83	<0.001	<0.001
CAVI	7.78	1.21	7.75	7.00	8.80	10.25	2.15	9.80	8.90	11.20	<0.001	<0.001
Weight (Kg)	83.2	15.6	81.55	73.40	93.50	68.27	15.68	65.90	57.10	80.00	<0.001	<0.001
Height (cm)	162.63	9.51	162.00	155.00	168.00	162.36	8.41	161.00	156.00	169.00	0.084	0.758
BMI (kg/m^2^)	31.38	4.82	31.15	28.20	34.60	25.84	5.68	25.40	21.50	30.30	<0.001	<0.001
Waist circumference (cm)	103.6	13.88	103.00	94.25	113.75	97.18	15.19	98.00	87.00	105.00	0.006	0.007
Glicemia (mg/dL)	99.5	23.34	93.00	86.00	106.00	103.6	38.68	93.00	82.75	109.50	0.396	0.980
Total cholesterol (mg/dL)	202.8	41.42	207.5	177.20	231.00	150.4	38.31	151.00	122.00	179.00	<0.001	<0.001
LDL cholesterol (mg/dL)	123.2	35.54	123.5	97.50	151.00	70.8	35.16	78.5	57.50	104.75	<0.001	<0.001
HDL cholesterol (mg/dL)	55.5	15.42	53.5	44.00	64.75	45.5	17.15	44.00	34.00	59.00	<0.001	<0.001
Triglycerides (mg/dL)	132.9	68.69	122.00	81.00	160.00	128.9	64.17	118.00	79.75	151.75	0.680	0.853
Creatinine (mg/dL)	0.86	0.19	0.84	0.72	0.98	1.05	0.58	0.92	0.73	1.14	0.005	0.078
	n	%				n	%				Chi-Square *p*	
Male sex	28	28				40	44				0.031	
Smoke	24	24				32	35				0.120	
Hypertension	62	62				71	78				0.024	
Diabetes	21	21				27	29				0.225	
Dyslipidemia	74	74				53	58				0.031	

*p*: *p*-value of the Student’s *t*-test; p*: *p*-value of the Mann-Whitney U test; SD: standard deviation, DBP: diastolic blood pressure; SBP: systolic blood pressure; CAVI: cardio-ankle vascular index; BMI: body mass index.

**Table 2:** Regression model considering CAVI0 as dependent variable, and age, DBP and the interaction between them as independent variables.

	Estimate	S.E.	t	*p*	p*
Intercept	−30.301	12.841	−2.360	0.019	0.012
Age	0.682	0.176	3.876	<0.001	<0.001
DBP	0.306	0.152	2.013	0.046	0.033
Interaction (age × DBP)	−0.005	0.002	−2.233	0.027	0.010

SE: standard error, DBP: diastolic blood pressure; p*: *p*-value of the permutation test.

**Table 3:** Regression model considering CAVI0 as dependent variable, and age, DBP, the interaction between them, sex and BMI as independent variables.

	Estimate	S.E.	t	*p*	p*
Intercept	−25.947	13.041	−1.990	0.048	0.032
Age	0.631	0.176	3.579	<0.001	<0.001
DBP	0.255	0.153	1.664	0.098	0.109
Interaction (age × DBP)	−0.005	0.002	−2.184	0.030	0.021
SBP	0.045	0.022	2.070	0.040	0.011
Male sex	0.712	0.685	1.039	0.300	0.473
BMI	−0.133	0.064	−2.084	0.039	0.032

SE: standard error, DBP: diastolic blood pressure; SBP: systolic blood pressure; BMI: body mass index; p*: *p*-value of the permutation test.

**Table 4:** Regression model considering CAVI as dependent variable, and age, DBP and the interaction between them as independent variables.

	Estimate	S.E.	t	*p*	p*
Intercept	−4.16	4.55	−0.914	0.362	0.253
Age	0.175	0.062	2.803	0.006	0.001
DBP	0.072	0.054	1.344	0.181	0.227
Interaction (age × DBP)	−0.001	0.001	−1.132	0.259	0.289

SD: standard error, DBP: diastolic blood pressure; p*: *p*-value of the permutation test.

**Table 5:** Regression model considering CAVI as dependent variable, and age, DBP, the interaction between them, SBP, sex and BMI as independent variables.

	Estimate	S.E.	t	*p*	p*
Intercept	−0.332	4.552	−0.073	0.942	0.572
Age	0.138	0.062	2.243	0.026	0.024
DBP	0.054	0.053	1.004	0.317	0.172
Interaction (age × DBP)	−0.001	0.001	−0.808	0.420	0.368
SBP	0.003	0.008	0.395	0.693	0.941
Male sex	0.533	0.239	2.228	0.027	0.012
BMI	−0.063	0.022	−2.828	0.005	0.018

SE: standard error, DBP: diastolic blood pressure; SBP: systolic blood pressure; BMI: body mass index; p*: *p*-value of the permutation test.

## Data Availability

The data presented in this study are available on request from the corresponding author.

## References

[1] Mitchell G.F., Parise H., Benjamin E.J., Larson M.G., Keyes M.J., Vita J.A., Vasan R.S., Levy D. (2004). Changes in Arterial Stiffness and Wave Reflection With Advancing Age in Healthy Men and Women. Hypertension.

[2] Miyoshi T., Ito H., Shirai K., Horinaka S., Higaki J., Yamamura S., Saiki A., Takahashi M., Masaki M., Okura T. (2021). Predictive Value of the Cardio-Ankle Vascular Index for Cardiovascular Events in Patients at Cardiovascular Risk. J. Am. Heart Assoc..

[3] Williams B., Mancia G., Spiering W., Rosei E.A., Azizi M., Burnier M., Clement D.L., Coca A., De Simone G., Dominiczak A. (2018). 2018 ESC/ESH Guidelines for Themanagement of Arterial Hypertension. Eur. Heart J..

[4] Shirai K., Utino J., Otsuka K., Takata M. (2006). A Novel Blood Pressure-Independent Arterial Wall Stiffness Parameter; Cardio-Ankle Vascular Index (CAVI). J. Atheroscler. Thromb..

[5] Spronck B., Avolio A.P., Tan I., Butlin M., Reesink K.D., Delhaas T. (2017). Arterial Stiffness Index Beta and Cardio-Ankle Vascular Index Inherently Depend on Blood Pressure but Can Be Readily Corrected. J. Hypertens..

[6] Shirai K., Suzuki K., Tsuda S., Shimizu K., Takata M., Yamamoto T., Maruyama M., Takahashi K. (2019). Comparison of Cardio–Ankle Vascular Index (CAVI) and CAVI0 in Large Healthy and Hypertensive Populations. J. Atheroscler. Thromb..

[7] Saiki A., Ohira M., Yamaguchi T., Nagayama D., Shimizu N., Shirai K., Tatsuno I. (2020). New Horizons of Arterial Stiffness Developed Using Cardio-Ankle Vascular Index (CAVI). J. Atheroscler. Thromb..

[8] Spronck B., Mestanik M., Tonhajzerova I., Jurko A., Jurko T., Avolio A.P., Butlin M. (2017). Direct Means of Obtaining CAVI 0—A Corrected Cardio-Ankle Vascular Stiffness Index (CAVI)—From Conventional CAVI Measurements or Their Underlying Variables. Physiol. Meas..

[9] Shirai K., Song M., Suzuki J., Kurosu T., Oyama T., Nagayama D., Miyashita Y., Yamamura S., Takahashi M. (2011). Contradictory Effects of Β1- and A1- Aderenergic Receptor Blockers on Cardio-Ankle Vascular Stiffness Index (CAVI). J. Atheroscler. Thromb..

[10] Shirai K., Shimizu K., Takata M., Suzuki K. (2017). Independency of the Cardio-Ankle Vascular Index from Blood Pressure at the Time of Measurement. J. Hypertens..

[11] Ibata J., Sasaki H., Kakimoto T., Matsuno S., Nakatani M., Kobayashi M., Tatsumi K., Nakano Y., Wakasaki H., Furuta H. (2008). Cardio-Ankle Vascular Index Measures Arterial Wall Stiffness Independent of Blood Pressure. Diabetes Res. Clin. Pract..

[12] Namekata T., Suzuki K., Ishizuka N., Shirai K. (2011). Establishing Baseline Criteria of Cardio-Ankle Vascular Index as a New Indicator of Arteriosclerosis: A Cross-Sectional Study. BMC Cardiovasc. Disord..

[13] Shirai K. (2011). Analysis of Vascular Function Using the Cardio–Ankle Vascular Index (CAVI). Hypertens. Res..

[14] Kiuchi S., Kawasaki M., Hirashima O., Hisatake S., Kabuki T., Yamazaki J., Ikeda T. (2015). Addition of a Renin-Angiotensin-Aldosterone System Inhibitor to a Calcium Channel Blocker Ameliorates Arterial Stiffness. Clin. Pharmacol..

[15] Nagayama D., Watanabe Y., Saiki A., Shirai K., Tatsuno I. (2019). Difference in Positive Relation between Cardio-Ankle Vascular Index (CAVI) and Each of Four Blood Pressure Indices in Real-World Japanese Population. J. Hum. Hypertens..

[16] Dobsak P., Soska V., Sochor O., Jarkovsky J., Novakova M., Homolka M., Soucek M., Palanova P., Lopez-Jimenez F., Shirai K. (2015). Increased Cardio-Ankle Vascular Index in Hyperlipidemic Patients without Diabetes or Hypertension. J. Atheroscler. Thromb..

[17] Nakamura K., Tomaru T., Yamamura S., Miyashita Y., Shirai K., Noike H. (2007). Cardio-Ankle Vascular Index Is a Candidate Predictor of Coronary Atherosclerosis. Circ. J..

[18] Kim K.J., Lee B.-W., Kim H., Shin J.Y., Kang E.S., Cha B.S., Lee E.J., Lim S.-K., Lee H.C. (2011). Associations Between Cardio-Ankle Vascular Index and Microvascular Complications in Type 2 Diabetes Mellitus Patients. J. Atheroscler. Thromb..

[19] Fantin F., Giani A., Trentin M., Rossi A.P., Zoico E., Mazzali G., Micciolo R., Zamboni M. (2022). The Correlation of Arterial Stiffness Parameters with Aging and Comorbidity Burden. J. Clin. Med..

[20] Bramwell J.C., Hill A.V. (1922). Velocity of transmission of the pulse-wave. Lancet.

[21] Saiki A., Sato Y., Watanabe R., Watanabe Y., Imamura H., Yamaguchi T., Ban N., Kawana H., Nagumo A., Nagayama D. (2016). The Role of a Novel Arterial Stiffness Parameter, Cardio-Ankle Vascular Index (CAVI), as a Surrogate Marker for Cardiovascular Diseases. J. Atheroscler. Thromb..

[22] Hayashi K., Yamamoto T., Takahara A., Shirai K. (2015). Clinical Assessment of Arterial Stiffness with Cardio-Ankle Vascular Index. J. Hypertens..

[23] Yamashina A., Tomiyama H., Takeda K., Tsuda H., Arai T., Hirose K., Koji Y., Hori S., Yamamoto Y. (2002). Validity, Reproducibility, and Clinical Significance of Noninvasive Brachial-Ankle Pulse Wave Velocity Measurement. Hypertens. Res..

[24] Spronck B., Mestanik M., Tonhajzerova I., Jurko A., Tan I., Butlin M., Avolio A.P. (2019). Easy Conversion of Cardio-Ankle Vascular Index into CAVI0. J. Hypertens..

[25] Kirkham F.A., Mills C., Fantin F., Tatsuno I., Nagayama D., Giani A., Zamboni M., Shirai K., Cruickshank J.K., Rajkumar C. (2022). Are You as Old as Your Arteries? Comparing Arterial Aging in Japanese and European Patient Groups Using Cardio-Ankle Vascular Index. J. Hypertens..

[26] Spronck B., Obeid M.J., Paravathaneni M., Gadela N.V., Singh G., Magro C.A., Kulkarni V., Kondaveety S., Gade K.C., Bhuva R. (2022). Predictive Ability of Pressure-Corrected Arterial Stiffness Indices: Comparison of Pulse Wave Velocity, Cardio-Ankle Vascular Index (CAVI), and CAVI0. Am. J. Hypertens..

[27] Giudici A., Khir A.W., Reesink K.D., Delhaas T., Spronck B. (2021). Five Years of Cardio-Ankle Vascular Index (CAVI) and CAVI0: How Close Are We to a Pressure-Independent Index of Arterial Stiffness?. J. Hypertens..

[28] Hung T.-J., Hsieh N.-C., Alizargar E., Bai C.-H., Wang K.-W.K., Hatefi S., Alizargar J. (2022). Association of Blood Pressure Indices with Right and Left Cardio-Ankle Vascular Index (CAVI) and Its Mathematically Corrected Form (CAVI0) for the Evaluation of Atherosclerosis. J. Pers. Med..

[29] Takahashi K., Yamamoto T., Tsuda S., Maruyama M., Shirai K. (2020). The Background of Calculating CAVI: Lesson from the Discrepancy Between CAVI and CAVI_0_. Vasc. Health Risk Manag..

[30] Webb A.J.S. (2020). Progression of Arterial Stiffness Is Associated With Midlife Diastolic Blood Pressure and Transition to Late-Life Hypertensive Phenotypes. J. Am. Heart Assoc..

[31] Franklin S.S., Gustin W., Wong N.D., Larson M.G., Weber M.A., Kannel W.B., Levy D. (1997). Hemodynamic Patterns of Age-Related Changes in Blood Pressure. Circulation.

[32] Liu X., Rodriguez C.J., Wang K. (2015). Prevalence and Trends of Isolated Systolic Hypertension among Untreated Adults in the United States. J. Am. Soc. Hypertens..

[33] Koracevic G., Stojanovic M., Kostic T., Lovic D., Tomasevic M., Jankovic-Tomasevic R. (2020). Unsolved Problem: (Isolated) Systolic Hypertension with Diastolic Blood Pressure below the Safety Margin. Med. Princ. Pract..

[34] Jurko T., Mestanik M., Jurko A., Spronck B., Avolio A., Mestanikova A., Sekaninova N., Tonhajzerova I. (2018). Pediatric Reference Values for Arterial Stiffness Parameters Cardio-Ankle Vascular Index and CAVI0. J. Am. Soc. Hypertens..

[35] Mestanik M., Jurko A., Spronck B., Avolio A.P., Butlin M., Jurko T., Visnovcova Z., Mestanikova A., Langer P., Tonhajzerova I. (2017). Improved Assessment of Arterial Stiffness Using Corrected Cardio-Ankle Vascular Index (CAVI_0_) in Overweight Adolescents with White-Coat and Essential Hypertension. Scand. J. Clin. Lab. Investig..

[36] Mills C.E., Govoni V., Faconti L., Casagrande M., Morant S.V., Crickmore H., Iqbal F., Maskell P., Masani A., Nanino E. (2020). A Randomised, Factorial Trial to Reduce Arterial Stiffness Independently of Blood Pressure: Proof of Concept? The VaSera Trial Testing Dietary Nitrate and Spironolactone. Br. J. Clin. Pharmacol..

[37] Mills C.E., Govoni V., Faconti L., Casagrande M.-L., Morant S.V., Webb A.J., Cruickshank J.K. (2017). Reducing Arterial Stiffness Independently of Blood Pressure. J. Am. Coll. Cardiol..

[38] Nagayama D., Fujishiro K., Suzuki K., Shirai K. (2022). Comparison of Predictive Ability of Arterial Stiffness Parameters Including Cardio-Ankle Vascular Index, Pulse Wave Velocity and Cardio-Ankle Vascular Index0. Vasc. Health Risk Manag..

[39] Plunde O., Franco-Cereceda A., Bäck M. (2021). Cardiovascular Risk Factors and Hemodynamic Measures as Determinants of Increased Arterial Stiffness Following Surgical Aortic Valve Replacement. Front. Cardiovasc. Med..

[40] Nagayama D., Fujishiro K., Miyoshi T., Horinaka S., Suzuki K., Shimizu K., Saiki A., Shirai K. (2022). Predictive Ability of Arterial Stiffness Parameters for Renal Function Decline: A Retrospective Cohort Study Comparing Cardio-Ankle Vascular Index, Pulse Wave Velocity and Cardio-Ankle Vascular Index0. J. Hypertens..

[41] Fantin F., Giani A., Gasparini L., Rossi A.P., Zoico E., Mazzali G., Zamboni M. (2021). Impaired Subendocardial Perfusion in Patients with Metabolic Syndrome. Diabetes Vasc. Dis. Res..

[42] Topouchian J., Labat C., Gautier S., Bäck M., Achimastos A., Blacher J., Cwynar M., De La Sierra A., Pall D., Fantin F. (2018). Effects of Metabolic Syndrome on Arterial Function in Different Age Groups: The Advanced Approach to Arterial Stiffness Study. J. Hypertens..

[43] Nagayama D., Sugiura T., Choi S.-Y., Shirai K. (2022). Various Obesity Indices and Arterial Function Evaluated with CAVI—Is Waist Circumference Adequate to Define Metabolic Syndrome?. Vasc. Health Risk Manag..

